# SWEET genes and TAL effectors for disease resistance in plants: Present status and future prospects

**DOI:** 10.1111/mpp.13075

**Published:** 2021-06-02

**Authors:** Pushpendra K. Gupta, Harindra S. Balyan, Tinku Gautam

**Affiliations:** ^1^ Department of Genetics and Plant Breeding CCS University Meerut India

**Keywords:** effectors, plant–microbe interactions, sugar transporters, SWEET, TALE

## Abstract

SWEET genes encode sugar transporter proteins and often function as susceptibility (S) genes. Consequently, the recessive alleles of these SWEET genes provide resistance. This review summarizes the available literature on the molecular basis of the role of SWEET genes (as S genes) in the host and corresponding transcription activator‐like effectors (TALEs) secreted by the pathogen. The review has four major sections, which follow a brief introduction: The first part gives some details about the occurrence and evolution of SWEET genes in approximately 30 plant species; the second part gives some details about systems where (a) SWEET genes with and without TALEs and (b) TALEs without SWEET genes cause different diseases; the third part summarizes the available information about TALEs along with interfering/truncated TALEs secreted by the pathogens; this section also summarizes the available information on effector‐binding elements (EBEs) available in the promoters of either the SWEET genes or the Executor R genes; the code that is used for binding of TALEs to EBEs is also described in this section; the fourth part gives some details about the available approaches that are being used or can be used in the future for exploiting SWEET genes for developing disease‐resistant cultivars. The review concludes with a section giving conclusions and future possibilities of using SWEET genes for developing disease‐resistant cultivars using different approaches, including conventional breeding and genome editing.

## Graphical abstract

SWEET genes function as susceptibility genes. They are activated by binding of TAL effectors (secreted by the pathogen) following a code and can be exploited for developing resistant cultivars.

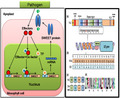

## 1 INTRODUCTION

Plant diseases in different crops cause an average global yield loss to the tune of approximately 16%, which can sometimes reach up to 40% to 50% in individual crops (Oerke, 2006; Savary et al., 2019). In view of this, the development of cultivars that are resistant to major diseases (including viral, bacterial, and fungal diseases) has been a priority area of research for crop breeding (Bailey‐Serres et al., 2019). During the last three decades (starting in the early 1990s), the molecular basis of plant immunity has also been extensively studied, and the genes imparting resistance have been identified, cloned, and characterized in many cases (Kourelis & van der Hoorn, 2018). As a result, the following two layers of plant immunity have been characterized: (a) pathogen‐/microbe‐/damage‐associated molecular pattern‐triggered immunity (PTI) and (b) effector‐triggered immunity (ETI). PTI and ETI are often explained on the basis of a zig‐zag model put forward by Jones and Dangl (2006), which is in conformity with a gene‐for‐gene model (Flor, 1942) according to which a plant with a resistance (R) gene will be resistant only when the prevalent pathotype of the pathogen carries a specific Avr gene. Modifications of this simple gene‐for‐gene model became available, which included the “guard model” and the “decoy model” (van der Hoorn & Kamoun, 2008; Paulus & van der Hoorn, 2018). On the basis of a meta‐analysis of 314 R genes that were already cloned in 2018, nine different mechanisms for disease resistance are known; it has also been shown that most R genes encode proteins with nucleotide‐binding site (NBS)–leucine‐rich repeat (LRR) domains (NLR proteins), but R genes encoding receptor‐like proteins/kinases are also known (Kourelis & van der Hoorn, 2018). A number of Avr genes from the pathogens causing diseases have also been cloned and characterized (Cai et al., 2002; Rouxel & Balesdent, 2010; Wang et al., 2017). Based on this knowledge, several recent reviews have been written, describing available strategies for developing disease resistance in crops (Kourelis & van der Hoorn, 2018; Sharma et al., 2019). However, in the majority of these reviews, information is missing about recessive resistance alleles of sensitivity genes like *Tsn1* in wheat and susceptibility (S) genes like Sugars Will Eventually be Exported Transporter (SWEET) genes in a number of crops, including rice (Jiang et al., 2020). The molecular basis of sensitivity genes like *Tsn1* and S genes like SWEET genes has also been elucidated, showing that entirely different mechanisms are involved in the two cases (Chen et al., 2010; Navathe et al., 2020).

In contrast to the above gene‐for‐gene model, the S genes follow an inverse gene‐for‐gene model, where the virulence/toxin gene of the pathogen can cause infection only when the host carries a dominant allele of a sensitivity/S gene. This system operates in the case of wheat diseases including tan spot, Stagonospora nodorum blotch, and spot blotch, where the host S gene like *Tsn1* is needed for the infection. The inverse gene‐for‐gene model also holds good in cases, where SWEET genes function as S genes. In such cases, SWEET genes are hijacked by the pathogen through its so‐called transcription activator‐like effectors (TALEs), which mimic transcription factors (TFs) to induce transcription of these SWEET genes, permitting one‐way transport of sugar to achieve susceptibility; this host–pathogen interaction model has been shown to hold good in a number of cases, including bacterial blight of rice (Chen et al., 2010; Verdier et al., 2012). The available literature on SWEET genes functioning as S genes and that on strategies to use SWEET genes for disease resistance in plants has not been adequately reviewed, although reviews are available on the pathosystem involving the bacterial pathogen *Xanthomonas oryzae* pv. *oryzae* (Xoo), which causes bacterial blight disease in rice (Doidy et al., 2012; Jiang et al., 2020; Vikal & Bhatia, 2017). The present review has been written to fill this gap. The review consists of four parts, which follow this brief introduction: The first part provides a brief review of the literature involving the occurrence of SWEET genes in approximately 30 plant species. The second part discusses details of the SWEET genes, which function as S genes in different plant species, including the model plant *Arabidopsis* and some crops (e.g., rice, cotton, cassava, pepper, grape). The third part deals with the molecular mechanism involved in host–pathogen interactions involving SWEET genes and/or TALEs; the code that is used by pathogens’ TALEs in binding to effector‐binding elements (EBEs) available in the promoters of SWEET genes will also be covered in this section. The fourth part of the review discusses possible approaches that are being used for developing resistant cultivars using SWEET genes. At the end of the review, conclusions are provided and manipulation of these SWEET genes to achieve disease resistance in crops is discussed.

## 2 SWEET GENES IN PLANTS

A genome‐wide survey of SWEET genes has been undertaken in >30 species (Table S1). The number of SWEET genes in these species varies from as low as 7 in the loquat fruit tree (*Eriobotrya japonica*; Wu et al., 2017) to as high as 108 in hexaploid wheat (*Triticum aestivum*; Gautam et al., 2019). Based on phylogeny, SWEET proteins in plants are classified into four clades. Clades I and II comprise SWEET proteins that transport hexose sugars (e.g., glucose and fructose), while proteins in the most common Clade III are specially meant for transport of sucrose. SWEETs in Clade IV mainly include vacuolar transporters that are involved in flux of fructose across the tonoplast (Chen et al., 2010, 2012). Some of the genes encoding SWEETs of Clade III also function as S genes because these proteins transport sucrose that is readily available for growth and development across the plasma membrane into the apoplast (Chen et al., 2012). Consequently, the recessive alleles of these SWEET genes can be deployed for developing resistant crop cultivars (see Feng & Frommer, 2015 for a review).

## 3 SWEET GENES FOR DISEASE RESISTANCE

**FIGURE 1:**
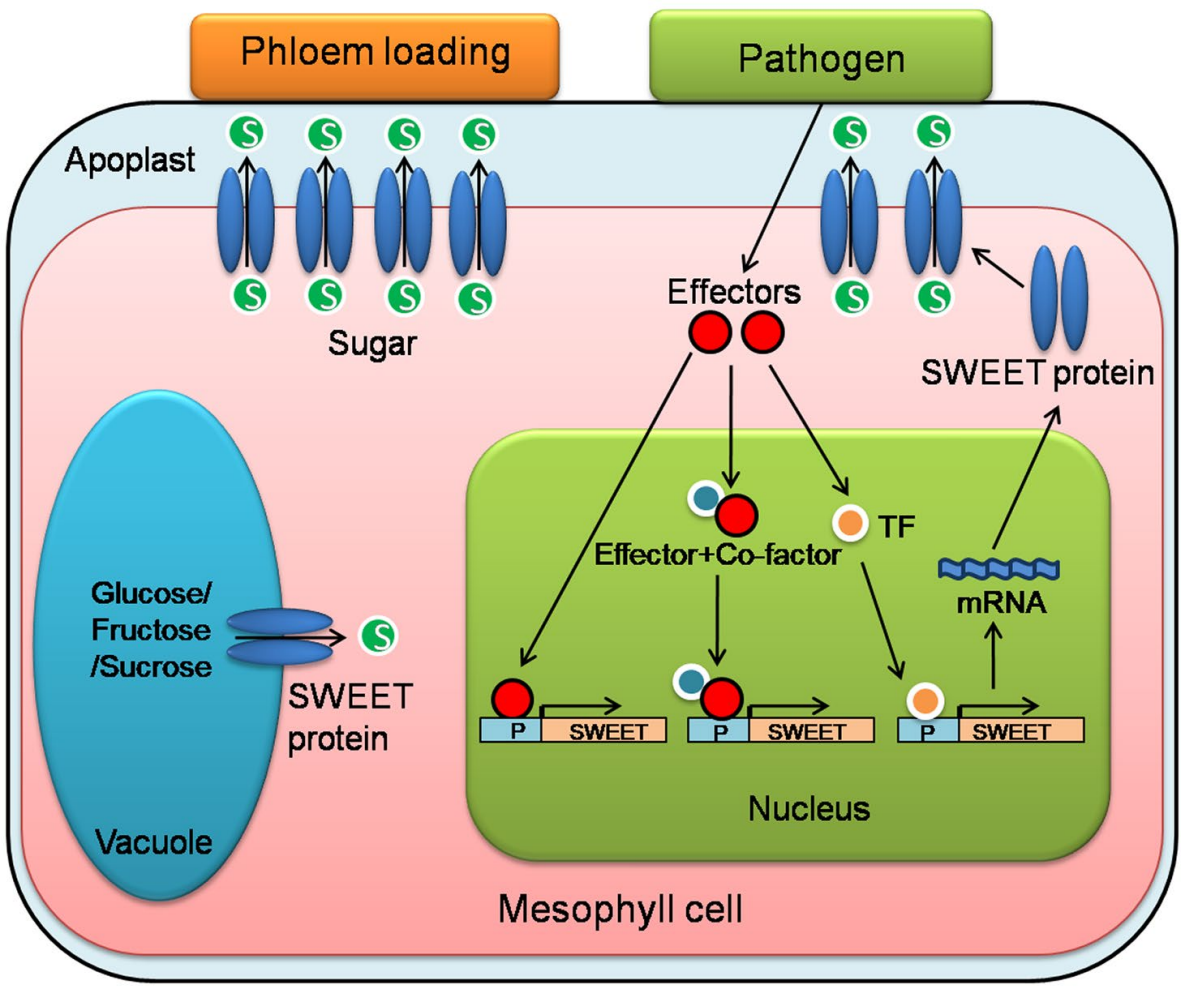
A simplified model for the role of plant SWEET sugar transporters in microbial nutrition. Upon infection, pathogens (e.g., *Xanthomonas oryzae* pv. *oryzae*) inject specialized effectors (TALEs) into the cytoplasm of host plant cells. On entering the nucleus, the TALEs induce the expression of host SWEET genes either directly or indirectly via the activation of transcription factors/cofactors, leading to the release of sugar into the apoplast as a source of nutrition for the pathogens

A number of SWEET genes that function as S genes and are exploited by bacterial as well as fungal pathogens are known (Tables 1 and 2). It has been hypothesized that SWEET genes are perhaps used by the pathogens for deriving nutrition for their growth and development, although the evidence in favour of such a hypothesis is not unequivocal and alternative hypotheses have been suggested (Bezrutczyk et al., 2018). It is also known that in the majority of cases where SWEET genes function as S genes, the pathogens secrete virulence proteins, described as TALEs, that target the promoters of SWEET genes of the host and activate their expression during infection to promote disease; the pathogens make use of the Type III secretion system (T3SS) for secreting these TALEs (Figure 1; Table 1; Bogdanove et al., 2010; Munoz‐Bodnar et al., 2013; Streubel et al., 2013; Verdier et al., 2012; Yu et al., 2011). Although bacterial blight of rice is the most important example where SWEET genes function as S genes, other examples of association of SWEET genes with diseases are now available in a number of other crops and the model plant species *Arabidopsis thaliana* (Tables 1 and 2). Some details of these pathogenesis‐related SWEET genes and other S genes used by the pathogens with and without the use of TALEs are described in this section. For convenience, these S genes will be described in three subsections.

**TABLE 1:** Host SWEET genes acting as susceptibility (S) genes and the corresponding bacterial effectors that facilitate disease development in different plant species

Host & SWEET gene	Bacterial pathogen; strain; disease	TALE protein	Reference
Rice: *OsSWEET11* ^1^ (*Os8N3*, *Xa13*)	Xoo; PXO99^A^; BB^2^	PthXo1	Talbot, 2010; Chen et al., 2010
Rice: *OsSWEET12* ^1^	Xoo; BB^2^	ArtTAL12^4^	Yang & White, 2004; Ochiai et al., 2005; Liu et al., 2011; Streubel et al., 2013; Li et al., 2013; Zhou et al., 2015
Rice: *OsSWEET13* ^1^ (*Xa25*)	Xoo; JXO1 & MAFF 311018; BB^2^	PthXo2/PthXo2.1/2.2	Zhou et al., 2015
Rice: *OsSWEET14* ^1^ (*Os11N3*, *Xa41*)	Xoo; PXO86/JXO1A/BAI3/ MAI1; BB^2^	PthXo3/ AvrXa7/TalC/TalF	Talbot, 2010; Streubel et al., 2013
Rice: *OsSWEET15* ^1^	Xoo; BB	ArtTAL15^4^	Streubal et al., 2013
*Arabidopsis*: *AtSWEET2*, *4*, *5*, *7*, *8*, *10* ^1^, *12* ^1^, *15* ^1^	(Pst)^2^	—	Chen et al., 2010; Streubel et al., 2013
Cotton: *GhSWEET10(c)* ^1^, D12_G18981, A12_G17471	Xcm; AR81009, H1005; BBC^3^	Avrb6^5^	Cox et al., 2017; Phillips et al., 2017
Cassava: *MeSWEET10a* ^1^	Xam; CBB^3^	TAL20_xam668_	Cohn et al., 2014
Pepper: *UPA 16*; *a*SWEET gene*	Xcv; 85‐10; BLS^3^	AvrBs3	Kay et al., 2009

^1^ SWEET genes for Clade III; BB, bacterial blight; BBC, bacterial blight of cotton; CBB, cassava bacterial blight; BLS, bacterial leaf spot.

^2^ hemibiotroph.

^3^ biotroph.

^4^ Artificial TALEs (or designer TALEs).

^5^ AvrB6 effector corresponding to the *GhSWEET10(c)* gene; —, no functional studies conducted and no TAL genes/TALEs identified so far.

* Correlative evidence linking SWEET gene expression induction to disease susceptibility; Xoo, *Xanthomonas oryzae* pv.*oryzae*; Pst, *Pseudomonas* *syringae* pv. *tomato*; Xcm, *X* *anthomonas* *citri* subsp. *malvacearum*; Xam, *Xanthomonas axonopodis* pv. *manihotis*; Xcv, *Xanthomonas* *campestris* pv. *vesicatoria*.

**TABLE 2:** SWEET genes that facilitate disease development due to fungal pathogens in different plant species (information based on correlative evidence linking SWEET gene expression induction to disease susceptibility)

Host & SWEET gene*	Fungal pathogen and disease	Reference
*Arabidopsis*: *AtSWEET11*, *15*	*Plasmodiophora brassicae*; clubroot disease^1^	Siemens et al., 2006; Chen et al., 2010; Li, Li, et al., 2018
*Arabidopsis*: *AtSWEET11*, *12*	*Golovinomyces cichoracearum*; powdery mildew	Chen et al., 2010
*Arabidopsis*: *AtSWEET2*, *8*	*Botrytis cinerea*; soft rot^2^	Veillet et al., 2017
Tomato: *SlSWEET15*	*Botrytis cinerea*; grey mold rot^2^	Asai et al., 2016
Grapevine: *VvSWEET4*, *7*, *15*	*Botrytis cinerea*; soft rot^2^	Chong et al., 2014; Breia et al., 2020
Banana: *MaSWEET‐4c*, *4d*, *14h*	*Fusarium odoratissimum* (*FOc TR‐4*); fusarium wilt^3^	Miao et al., 2017
Wheat: *TaSWEET2b*, *5a*, *14g*, *14i*	*Puccinia graminis*; stem rust^1^	Gao, Wang, et al., 2018
*Brassica rapa*: *BrSWEET1a*, *2a*, *2b*, *9*, *11a*, *14c*, *15b*, *15c*, *16a*, *17*	*P. brassicae*; clubroot^4^	Li, Li, et al., 2018
*Brassica oleracea*	*P. brassicae*; clubroot	Zhang et al., 2019
Rice: *OsSWEET11*	*Rhizoctonia solani*; sheath blight^2^	Gao, Zhang et al., 2018

^1^ obligate biotroph.

^2^ necrotroph.

^3^ hemibiotroph.

^4^ soilborne biotroph.

* Correlative evidence linking SWEET gene expression induction to disease susceptibility.

### 3.1 SWEET genes hijacked by pathogens using TALEs

The most common examples of SWEET genes that are up‐regulated by TALEs include those identified in rice, cotton, and cassava (Table 1). Among the three rice SWEET genes that have been characterized, the gene *OsSWEET11* (*Xa13*), which provides resistance against bacterial blight caused by *X*. *oryzae*, as well as sheath blight caused by *Rhizoctonia solani*, is most important, although the functional mechanisms differ (see next section). The role of this gene in susceptibility to sheath blight was confirmed by the reduced susceptibility of transgenic plants carrying the mutant allele *Ossweet11* and the increased susceptibility due to overexpression of the dominant allele *OsSWEET11* (Gao, Zhang, et al., 2018).

Another interesting example of a SWEET gene functioning as an S gene is *UPA16* in pepper (UPA stands for “up‐regulated by AvrBs3”, the founder member of the TALE family), whose expression is induced by AvrBs3 in response to attack by *Xanthomonas campestris* pv. *vesicatoria* (Kay et al., 2009), which causes the disease bacterial leaf streak (for details see Table 1). However, the role of *UPA16* as a SWEET gene in bacterial leaf streak in pepper has not been studied in detail. The other UPA genes that have been identified in pepper and whose expression is induced by AvrBs3 are not SWEET genes (see Section 3.3).

### 3.2 SWEET genes used by plant pathogens without using TALEs

A summary of SWEET genes that facilitate disease development by fungal pathogens without involving TALEs is provided in Table 2. These genes include five genes involved in fungal diseases of *Arabidopsis* and the genes causing clubroot disease of *Brassica rapa* and *Brassica oleracea*. Clubroot is a major disease in crops belonging to the above two *Brassica* species and is caused by the soilborne fungus *Plasmodiophora brassicae*. Using a pair of near‐isogenic lines, including a clubroot‐resistant (CR) and a clubroot‐susceptible (CS) line for the clubroot resistance gene *CRb* in Chinese cabbage (*B*. *rapa*), it was shown that infection by the pathogen led to a significant increase of glucose and fructose content in the roots of the CS line relative to that in the CR line. In another study involving field mustard (*B*. *oleracea*), higher expression levels were observed for six BoSWEET genes in roots of a CS cabbage cultivar (CS‐JF1) at 7 days after inoculation. Localization of a subset of BoSWEET proteins in the plasma membrane was also confirmed in CS‐JF1 (Zhang et al., 2019). These studies suggest that infection by *P*. *brassicae* triggers active sugar translocation between the sugar‐producing tissues and the clubbed tissues. It was inferred that SWEET genes act as S genes, facilitating growth of the pathogen (Li, Li, et al., 2018; Zhang et al., 2019).

### 3.3 Alternatives for SWEET genes used by TALEs

Pathosystems with TALEs having targets other than SWEET genes are also known (Table S2). Some representative examples are briefly described.

The rice gene *Xa5*, which is the dominant allele of the widely deployed R gene *xa5*, encodes TFIIAγ5 (a small subunit of the basal TF TFIIA), which is involved (as a cofactor) in the activation of S genes; its recessive allele *xa5* encodes a protein that carries a single amino acid substitution (V39E) in TFIIAγ5, thus providing resistance against bacterial blight. Another unknown gene encodes TFIIAγ1, which compensates for the absence of TFIIAγ5, but no corresponding R gene like *xa5* is known for TFIIAγ1 (Jiang et al., 2006; Ma et al., 2018; Mücke et al., 2019).

*OsTFX1* (encoding a basic leucine zipper TF) is activated by the effector PathXO6 and the gene encoding TFIIAγ1 (a small subunit of TFIIA) is activated by the effector PathXO7; the genes encoding both these effectors are carried by PXO99^A^ and cause bacterial blight.

*CsLOB1* is the target gene for citrus bacterial canker in citrus fruit, which is caused by *Xanthomonas citri* pv. *citri* and *X*. *citri* pv. *aurantifolii*. The expression of this gene is induced by each of three TALEs (PthA4, PthA_W_, and PthA*) from *X. citri* pv. *citri* and also by PthB and PthC, which are associated with susceptibility to citrus canker (Ference et al., 2018; Hu et al., 2014). Mutations in the EBE domain of the promoter region of the S gene *CsLOB1* have been developed and used for resistance; in particular, the gene editing approach involving CRISPR/Cas9 has been found to be effective (Jia et al., 2016, 2017; Peng et al., 2017).

The S gene *TaNCED‐5BS* (which encodes 9‐*cis*‐epoxycarotenoid dioxygenase), involved in bacterial streak in wheat, which is caused by *Xanthomonas* *translucens* pv. *undulosa*, is another example where the corresponding TALEs bind to the promoter of the gene. *TaNCED‐5BS* encodes the enzyme that catalyses the rate‐limiting step in abscisic acid (ABA) biosynthesis and has been shown to be responsible for susceptibility to this disease.

A number of UPA genes in pepper (except *UPA16*, which is a SWEET gene, and *UPA20*, which encodes a bHLH TF) are activated by a TALE (AvrBs3), which binds to the UPA box available in the promoter of each of these genes (Kay et al., 2007, 2009; Romer et al., 2007, 2009). The proteins encoded by these UPA genes are included in Table S2 (also see table 1 in Kay et al., 2009).

## 4 TALES TARGETING SWEET GENES

**FIGURE 2:**
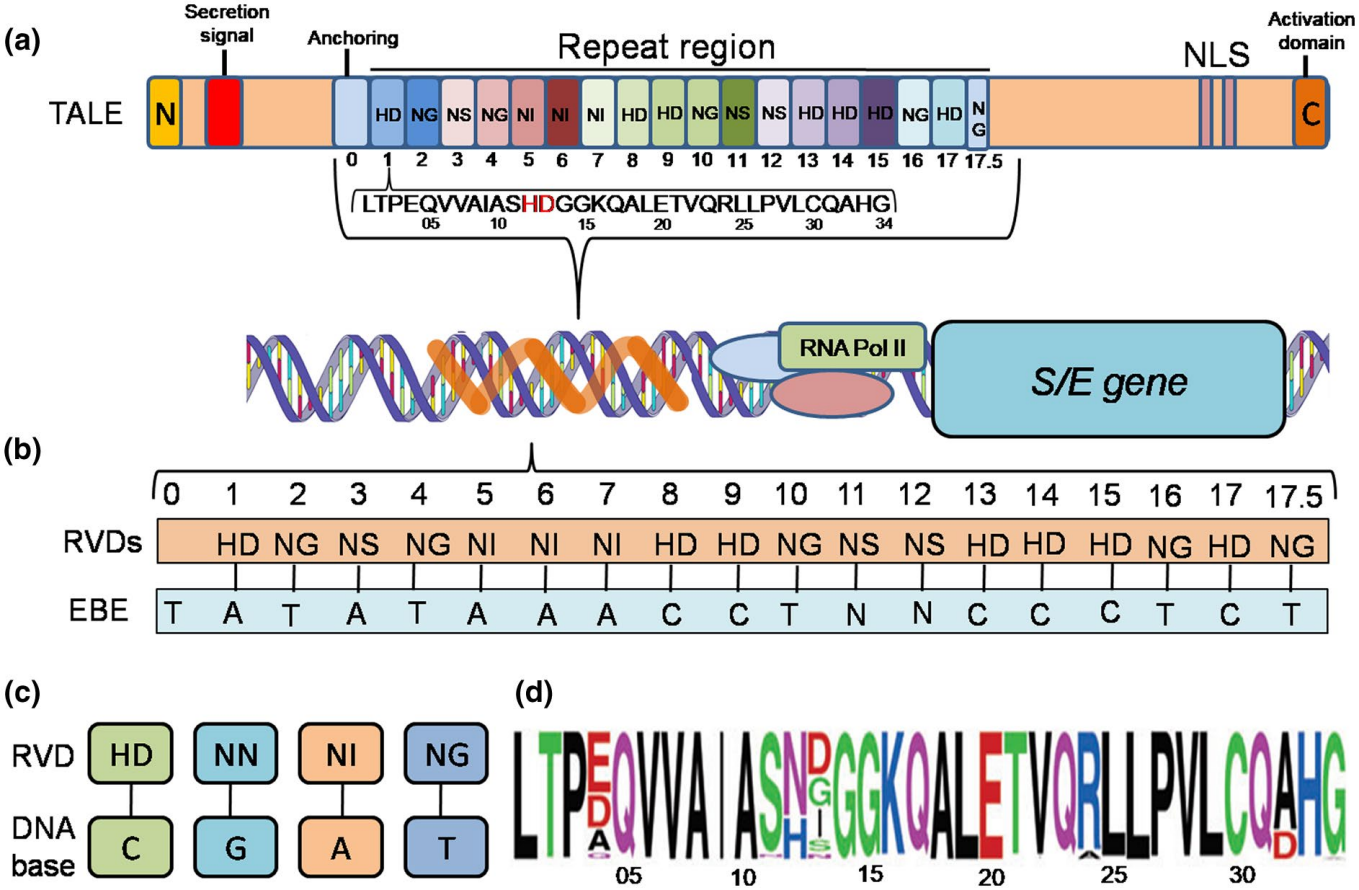
Specificity of TALEs for the binding elements in target DNA (promoters of SWEET genes). (a) A TALE (AvrBs3) showing the following features: (i) a N‐terminal end, (ii) central tandem repeats, (iii) a C‐terminal end containing NLS and AD domains. Next is shown a central repeat with its 34 amino acids, with those at positions 12 and 13 being hypervariable amino acids representing a repeat variable diresidue (RVD) (HD highlighted) that is available in each repeat and is involved in reading the target effector‐binding element (EBE) according to the code, which has already been discovered. (b) Schematic representation of the interaction between the RVDs and the bases in the EBE. (c) A minimal set of four RVDs and the corresponding DNA bases following the code. (d) A positional weight matrix (PWM) as WebLogo showing variation in the RVD (at positions 12 and 13) and other positions in a 34‐amino acid long repeat. NLS, nuclear localization signal; AD, activation domain

For a number of diseases, the pathogen delivers a variety of Type III effectors (T3Es) through the T3SS into the host cell, so that TALEs represent a special class of these T3Es (Muñoz‐Bodnar et al., 2013; Ponciano et al., 2003). These T3Es bind to the promoter sequences of a variety of genes (including SWEET genes) with the help of its central repeat region, which carries a variable number of repeats, generally ranging from 1.5 to 33.5 (Boch & Bonas, 2010); of these, AvrBs3 (17.5) and AvrHah1 (13.5), each binding the promoter of the pepper *Bs3* R gene, have been studied in some detail. Each TALE repeat, in turn, is 33–35 amino acids long. A typical repeat sequence is LTPEQVVAIASHDGGKQALETVQRLLPVLCQAHG. Each TALE also carries a C‐terminal segment with a nuclear localization signal (NLS) domain and an activation domain (AD) (Boch et al., 2014). Also, in each repeat, at positions 12 and 13 (HD in the above case), there is a repeat variable diresidue (RVD) that binds a specific DNA base in the binding site available in the promoter of the target gene. The binding site is the EBE in the case of SWEET genes and the UPA box in the case of UPA genes (Boch et al., 2009; Moscou & Bogdanove, 2009). X‐ray crystallography analysis revealed that a TALE protein consists of a right‐handed superhelix that winds around the major groove of double‐stranded DNA, interacting with the bases of the sense strand (Figure 2b).

### 4.1 TALE‐EBE code (a protein–DNA code for binding)

The TALE (protein)‐EBE (DNA) code was resolved in 2009 by two independent groups. The first group, led by Adam Bogdanove, searched for patterns of alignment between amino acid sequences of TALE proteins and nucleotide sequences of target promoters of SWEET genes using a database of genes up‐regulated by TALEs (Moscou & Bogdanove, 2009). The second group, led by Jens Boch, deduced the code through molecular analysis of the most important TALE, AvrBs3 (Figure 2a), and its target DNA sequence in the promoter of a pepper gene like *B3* (this gene is a UPA gene, which may not be a SWEET gene) that is activated by AvrBs3 (Boch et al., 2009). The experimentally validated code between the RVD (a pair of amino acids) and the DNA base in the EBE can be expressed as follows: (a) NI: A; (b) HD: C; (c) NG: T or ^5^mC; (d) N: R; (e) NS: N; (f) NK or NH: G (where N may be any base; also shown in Figure 2c). It has been shown that the amino acid at position 13 determines the base with which the RVD binds and the amino acid at position 12 performs a general subsidiary role. It has also been shown that the RVD in the form of NS and a deletion of the amino acid residue at position 13 (N*) have no nucleotide specificity and can bind any base in the EBE (an asterisk indicates the lack of an amino acid at the base‐specifying residue). It is apparent that with the availability of 20 amino acids, the number of possible RVDs would be 400, but only a few are predominantly found in TALEs. It has also been shown that different pathogens may use different versions of the TALE‐EBE code with distinct sets and frequencies of RVDs (for details, see the review by Schornack et al., 2013).

The discovery of the TALE‐EBE code provided opportunities for the in silico discovery of the TALE genes in pathogens and EBEs in the promoters of the target genes (generally SWEET genes). The code has also been used for developing designer TALEs (dTALEs) that have been used to activate or repress the expression of SWEET genes and to explore their functions (Miller et al., 2011; Noel et al., 2013). There are also reports that focused on in silico prediction of EBEs in plant genomes as targets of TALEs with known structure (Grau et al., 2013; Moscou & Bogdanove, 2009; Pérez‐Quintero et al., 2013).

### 4.2 Bioinformatics tools for the code

A number of bioinformatics tools (webserver interphase and/or stand‐alone software) are also available, which can predict the amino acid sequence of the central repeat region of TALEs and the nucleotide sequences of EBEs that are available in the promoters of host SWEET genes. These tools and their essential features are summarized in Table S3. Five important tools (besides several others) include Target Finder, Talvez, Storyteller, TALgetter, and PrediTALE. Each of these five tools usually allows identification and prediction of EBE targets for almost all known TALEs, although PrediTALE (see below) has been shown to be superior to other tools in terms of the prediction of true positives (Erkes et al., 2019). Two other tools, namely DisTAL and FuncTAL, are available in the QueTAL suit; these programs also allow classification of TALEs according to phylogeny and similarity of DNA‐binding specificities. Another important tool is AnnoTALE, in which the nomenclature of TALEs makes use of different classes of TALEs and the origin of the strain of the TALEs within a class. Another important program, TargeTALE, allows identification of targets of each TALE with high prediction efficiency. It has also been recognized that each of these algorithms sometimes also gives false positives and false negatives. Therefore, a continuous revision and improvement of algorithms used in bioinformatic approaches is necessary (Erkes et al., 2019; Noel et al., 2013). In addition to the tools, a database for TALE‐related data of the rice–Xoo system (daTALbase) has also been developed, which allows the study of the variations in TALEs and EBEs for the rice–Xoo pathosystem (Pérez‐Quintero et al., 2018). A diagnostic kit for bacterial blight of rice with a number of tools has also been recently developed (Eom et al., 2019; see Section 5 for more details about this kit).

### 4.3 Artificial TALEs or dTALEs

Utilizing the TALE‐EBE code described above, it was also possible to develop artificially designed TALEs (dTALEs) for specific EBEs; these dTALEs may be delivered through the bacterial secretion system to activate the host genes for susceptibility/resistance or for the purpose of their functional analysis. For developing dTALEs, modification of only the RVD is required; non‐RVD sequences have no major effect on binding to EBEs (Morbitzer et al., 2010). The dTALEs could also be used to determine whether or not induction of expression of specific SWEET genes is essential for pathogenicity, as demonstrated for the *GhSWEET10* gene for susceptibility to bacterial blight in cotton (Cox et al., 2017). A dTALE has also been used to induce expression of a recessive R gene, *xa13*, which is an allele of the S gene *Os8N3*/*Xa13*/*OsSWEET11* in rice (*Oryza sativa*); *Xa13* rendered the resistant rice cultivar IRBB13 (*xa13/xa13*) susceptible to Xoo (Li et al., 2013). Thus, utilizing this approach, it is possible to expand the repertoire of SWEET S genes in rice, cotton, and cassava (Cohn et al., 2014; Streubel et al., 2013).

### 4.4 Interfering/truncated TALEs: NLR resistance genes *Xa1*/*Xo1*

In the nucleus, TALEs either directly bind to EBEs available in the promoters of host S genes (e.g., SWEET genes *Xa13*, *Xa25*, and *Xa41*), causing infection, or interact with R genes (e.g., Executor R [ER] genes *Xa10*, *Xa23*, and *Xa27*), providing resistance. In rice, the *Xa1* gene is responsible for resistance against all strains of Xoo that otherwise cause bacterial blight; *Xo1* is a similar gene for resistance against both bacterial blight caused by Xoo and bacterial leaf streak caused by *X. oryzae* pv. *oryzicola* (Xoc) and was discovered later (Triplett et al., 2016). Both these genes provide resistance due to interaction with TALEs.

The resistance due to genes *Xa1* and *Xo1* can be overcome by the pathogen through secretion of interfering/truncated TALEs (iTALEs/truncTALEs), which have been discovered during the last 5 years (Ji et al., 2016; Read et al., 2016). iTALEs/truncTALEs are encoded by genes that were earlier believed to be pseudogenes (Ji et al., 2016). iTALEs are classified into the following two groups: type A iTALEs, which are characterized by the C‐terminal truncation of 103 amino acids, due to the occurrence of a premature stop codon introduced by a C‐to‐T change in the coding sequence of the gene; and type B iTALEs, which are characterized by a loss of 229 amino acids due to mutation in the 3′‐end of the gene. These iTALEs act as effector decoys to prevent NLR activation of *Xa1*, leading to susceptibility to bacterial blight (Paulus & van der Hoorn, 2018; Paulus et al., 2017). The mechanism of action of iTALEs in suppressing the resistance due to *Xa1* is still unknown but it seems not to interfere with the activation of *Xa1* transcription (Ji et al., 2016). The evolution of these new decoy effectors is a novel virulence strategy to achieve effector‐triggered susceptibility (ETS) in the host plant (Cummin & Huitema, 2017).

The discovery of iTALEs for suppression of the activity of the R gene *Xa1* involved a study of a large number of deletion mutants generated in a unique pathogen strain, PXO99^A^, which is virulent against almost all rice cultivars (Ji et al., 2016); the genome of PXO99^A^ was sequenced by Salzberg et al. (2008) and was found to be much longer than those of other strains. Its genome carries 87 additional genes that are absent in two other strains that were examined. Ji et al. (2016) conducted a study to understand the function of each of the 19 TALEs (organized in nine clusters) carried by this strain. The 19 TALEs carried by PXO99^A^ also included three erstwhile pseudogenes, which were later found to be functional genes during mutation studies conducted by Ji et al. (2016). The majority of mutants with deletion of the PthXo1 cluster of genes encoding TALEs lost their ability to cause the disease in susceptible rice varieties, but two deletion mutants, namely TAL13a (with a stop codon causing a deletion of 103 amino acids at the C‐terminal end) and TAL13b (with a deletion of 688 bp), showed resistance in two rice varieties (IRBB1 and Kogyoku) but not in other rice lines susceptible to PXO99^A^. This inability of the iTALE gene to suppress *Xa1* resistance in IRBB1 and Kogyoku to some Xoo strains needs further investigation (Ji et al., 2016).

In a subsequent study on the gene *Xa1*, Ji et al. (2020) cloned and characterized five additional R genes that are allelic to *Xa1*: *Xa2*, *Xa31*(*t*), *Xa14*, *CGS‐Xo1*_*11*_, and *Xa45*(*t*). It was shown that all rice plants carrying *Xa1*, including transgenic rice plants carrying *Xa1*, were resistant to all such Xoo strains that lacked genes encoding iTALEs.

The *Xo1* gene for resistance against bacterial leaf streak was also studied in parallel by Read et al. (2016, 2020) in the rice variety Carolina Gold Select, and was found to be located within a genomic region carrying 14 NLR type R genes. It was hypothesized that *Xo1* is one of these 14 NLR genes, and was designated as *CGS‐Xo1_11_*, which is allelic to *Xa1* (Read et al., 2020).

### 4.5 Executor R genes and TALEs

The ER genes represent a family of genes that provide resistance by recognizing the cognate TALEs derived from the pathogen. These ER genes differ from the classical R genes, because the specificity of these R genes does not lie in the coding sequence of the R gene but in the site for interaction with the cognate TALE. The specificity of TALE (for a specific ER gene) depends on the NLS domain of its C‐terminal region and the central repetitive transcription AD described earlier in this review. A TALE binds to an EBE available in the promoter of an ER gene, inducing transcription of this gene for providing resistance. Thus, a TALE may either bind to an EBE in a SWEET gene, causing susceptibility, or bind an available ER gene, providing resistance. While inducing ER gene expression, a TALE behaves like an Avr gene, suggesting a gene‐for‐gene relationship between TALE genes in the pathogen and ER genes in the host. Five ER genes have so far been cloned: *Xa27*, *Bs3*, *Bs4C‐R*, *Xa10*, and *Xa23*. Another uncharacterized ER gene is *Xa7*, which is targeted by AvrXa7.

## 5 MANIPULATION OF SWEET GENES FOR DISEASE RESISTANCE

SWEET genes provide only one of the several systems for disease resistance available to plant breeders. One possible approach to achieve resistance is to utilize the system (in the host) that prevents or reduces sugar supply. This mechanism has been described as “sugar starvation for disease resistance” or “starvation‐mediated resistance” (Oliva & Quibod, 2017). However, it may be better described as “resistance by loss of susceptibility”. The majority of approaches for manipulation of SWEET genes are based on this hypothesis and are briefly described in this section. Results of some studies undertaken to achieve disease resistance using resistance by loss of susceptibility are summarized in Table S4.

### 5.1 Control of activity of TALEs for disease resistance

Four different possibilities to control the activity of TALEs were suggested by Schornack et al. (2013). More details of these four possibilities, including figures and examples with relevant references, are available in the review by Schornack et al. (2013). Some examples of using these approaches are also listed in Table S4, which may overlap the above four approaches. However, the utility of these four approaches by plant breeders in developing resistant cultivars is yet to be demonstrated.

### 5.2 Sequence variations in promoters of SWEET genes

To achieve resistance to diseases caused due to SWEET S genes, breeders may also exploit genetic variability that may occur in promoters of SWEET genes. These variations may be natural or artificially induced using mutagenesis. Some of these variations may prevent the binding of TALEs to EBEs, so that the concerned SWEET gene(s) will not be activated. The majority of such mutants have been shown not to have any negative effect on plant phenotype, thus making these mutants useful for developing resistant cultivars (Chen et al., 2010). This phenomenon may be illustrated using several available examples including variation in the promoters of *OsSWEET13* (*xa25*) and *OsSWEET14* (*xa41*), which are direct targets of the following five major TALEs: PthXo2, PthXo3, AvrXa7, TalC, and TalF (Zaka et al., 2018). Sometimes, the same promoter can also have more than one independent EBE for binding of more than one TALE; such a situation can be anticipated in the case of *xa13*, which is involved in resistance against both bacterial blight and sheath blight in rice; in such cases, breeders will have to search for variability in all available EBEs. Some of the rice EBE variations that disallow binding of TALEs are summarized in Table S5. Variations have also been reported in promoter segments other than EBEs (Hutin et al., 2015; Ji et al., 2018; Liu et al., 2011).

The above individual variations providing resistance may also be pyramided and used with other R genes to provide durable and broad‐spectrum resistance against bacterial blight in rice. Deletion of the gene encoding the effector PthXo1 in the pathogen and silencing of *OsSWEET11* in the host both lead to a reduction in disease severity when challenging the host with the pathogenic strain PXO99^A^. It is also known that indica rice IR24 carrying *OsSWEET13* is susceptible to Xoo strains expressing PthXo2 (a TALE). Heterologous expression of *OsSWEET13* in *Nicotiana benthamiana* leaf cells also elevated sucrose concentrations in the apoplast, thus supporting the conclusion that Xoo enhances the release of sucrose from host cells, helping the pathogen in the compatible interaction. However, the gene *OsSWEET13* of two japonica rice cultivars, namely Nipponbare and Kitaake, lack EBEs for PthXo2, so that these two cultivars are resistant to this strain (Zhou et al., 2015). This disease susceptibility in IR24 was shown to be associated with a solitary single‐nucleotide polymorphism (SNP); an alternative allele of this SNP has been shown to be responsible for resistance that is associated with *xa25* (derived from cv. Minghui). Using this information, it was also possible to use gene editing involving CRISPR/Cas9 technology to achieve resistance through mutation in the causal SNP in the promoter of the S gene *OsSWEET13* (Oliva et al., 2019; also see later for more details).

As mentioned above, the expression of *OsSWEET13* is a prerequisite for susceptibility to Xoo strains secreting the TALE PthXo2. In a survey, it was observed that only 42 of 104 Xoo strains could induce expression of *OsSWEET13* in a particular genotype, suggesting that the target genotype is susceptible to these 42 strains only and is resistant to the remaining 62 strains (Zhou et al., 2015). Therefore, information about the prevalent strain of the pathogen may also be used for breeding a resistant cultivar.

Naturally occurring alleles for resistance for a particular disease may also be identified by allele mining using the approach of EcoTILLING, if the sequences of the SWEET gene are available (e.g., 3K sequences in rice). In this context, one can use computational approaches to allow identification of genes carrying EBEs in the dominant SWEET alleles’ promoters. Once validated, the EBEs provide an opportunity for finding insensitive promoters, as in the case of *xa13*. This approach was successfully used by Comai et al. (2004). In addition, TILLING may also be used for artificial induction and allele mining for variation in the EBEs in the promoters of the dominant alleles of SWEET S genes. This could lead to identification of variants in EBEs that render them incompatible for the binding of TALEs, thus providing resistance against the pathogens. No examples of using TILLING to achieve SWEET gene‐mediated resistance are available so far, but the approach can certainly be used in the future. Examples of the successful use of TILLING are available for developing *mlo*‐based resistance against powdery mildew in wheat (Acevedo‐Garcia et al., 2017).

### 5.3 Genome editing of SWEET genes for resistance

Two important tools for genome editing include TALE nucleases (TALENs) and CRISPR/Cas9; both have been used successfully for the modification of EBEs in the promoters of SWEET genes (Table S3).

#### 5.3.1 TALEN‐mediated genome editing

There are at least two studies involving TALEN‐mediated genome editing of SWEET genes, including *OsSWEET11* and *OsSWEET14* (Blanvillain‐Baufumet et al., 2017; Li, Liu, et al., 2012). Of these two genes, the gene *OsSWEET14* is an unusual SWEET S gene, because it is the target of at least four TALEs, namely AvrXa7, PthXo3, TLC, and TLF (Table 1). In the first of the above two studies, Li, Liu, et al. (2012) obtained TALEN‐mediated mutations in the promoters of both genes (*OsSWEET11* and *OsSWEET14*), but involving binding sites for only two of the four TALEs (AvrXa7 and PthXo3) in the case of *OsSWEET11*. The mutations obtained in this study provided resistance against the relevant strains of the pathogen. In the other study, Blanvillain‐Baufumet et al. (2017) edited *OsSWEET14* against all four TALEs mentioned above. They generated an allele library of the *OsSWEET14* promoter through dTALENs. Plants edited for EBEs used by TalF (AvrXa7), but not those used by TalC, were resistant to bacterial strains. The TalC EBE mutant line was, however, resistant to a strain expressing a dTALE inducing *OsSWEET14* expression, whose EBE was also edited in this line. It was speculated that TalC may have additional targets, suggesting that TALE‐mediated plant susceptibility may result from induction of several, genetically redundant, host S genes by a single effector.

#### 5.3.2 CRISPR/Cas9‐mediated genome editing

Two studies involving CRISPR/Cas9‐mediated genome editing of EBEs were published in 2019 (Oliva et al., 2019; Xu et al., 2019). Both studies involved the use of the popular rice cv. Kitaake, although two additional mega cvs., IR64 and Ciherang‐Sub1, were also used by Oliva et al. (2019); they used CRISPR/Cas9‐mediated genome editing to achieve resistance against a collection of 94 Xoo strains of Asian origin. The rice cv. Kitaake was also used in other studies, because it has a rapid life cycle (9 weeks seed to seed) and is easy to transform and propagate. Kitaake is also known to harbour the recessive R allele of *OsSWEET13* (*xa25*), so that Xu et al. (2019) initially used only the remaining two genes (*OsSWEET11* and *OsSWEET14*) for editing, leading to the production of an edited genotype named MS14K, which carried broad‐spectrum bacterial blight resistance. When MS14K was tested using 131 Xoo strains, it was found to be resistant to only 121 strains and susceptible to 10 Xoo strains, suggesting that these 10 Xoo strains may carry novel TALEs that activate S genes. Therefore, MS14K was used once again for editing the third gene (*OsSWEET13*). Xu et al. (2019) also examined the whole genome sequences of 3,000 rice genotypes to study EBE variations in the promoter of *OsSWEET13* that was already present in Kitaake. Oliva et al. (2019), moreover, examined genome sequences of 69 Xoo strains in order to identify multiple TALE variants that could bind to the promoters of *OsSWEET13* alleles.

The objective of both studies was to achieve durable broad‐spectrum resistance against bacterial blight through editing of EBEs for all the three SWEET genes (*OsSWEET11*, *13*, and *14*); this objective was successfully achieved by both studies, although it remains unclear whether the edits introduced in the two studies are different. It is likely that there are some minor differences leading to the same result in terms of failure of binding of all known TALEs to the promoters of the three genes. One may also wonder whether it is desirable to combine the edits obtained by the two groups to further enhance the level of durable broad‐spectrum resistance against bacterial blight.

A diagnostic kit has also been recently developed to facilitate studies on resistance against bacterial blight and to identify resistant lines in the field (Eom et al., 2019). The kit carries a SWEET promoter database along with primers for reverse transcription PCR to identify SWEET genes in the host and TALE genes in the pathogen. Therefore, it is possible for breeders to select an optimal gene set for resistance in a particular geographical region and to breed accordingly.

### 5.4 Promoter trap and Executor R genes

A brief account of ER genes is presented in Section 4.5. The proteins encoded by these genes share no sequence similarity with proteins of known function. ER genes can be classified into two groups: Group 1, including genes encoding proteins with a probable function in plant development, but whose function has been hijacked by the host for adaptation to disease resistance (Exposito‐Rodriguez et al., 2011; Römer et al., 2007); and Group 2, including genes encoding relatively short proteins with membrane‐spanning domains having multiple hydrophobic potential. Three known dominant Xa genes (*Xa10*, *Xa23*, and *Xa27*) for resistance against bacterial blight represent these Group 2 ER genes, each containing multiple transmembrane domains functioning as a promoter trap (Gu et al., 2005; Liu et al., 2011; Tian et al., 2014; Wang et al., 2015; Zhang et al., 2015). For instance, the dominant R gene *Xa27* of rice is an ER gene that carries a binding site for an Xoo TALE (AvrXa27) in its promoter (Gu et al., 2005; Zhang et al., 2015). Increased expression of *Xa27* results in thickened vascular bundle elements, even in the absence of Xoo infection. Rice lines expressing Xa27 show enhanced resistance when inoculated with compatible strains of Xoo and Xoc (Tian & Yin, 2009). The other two rice genes, *Xa10* and *Xa23*, which confer resistance against Xoo, have also been found to be ER genes (Tian et al., 2014; Wang et al., 2015). EBEs can also be used by the host plant to generate promoter traps, which could recruit TALEs and thus mediate expression of ER genes (Schornack et al., 2013).

### 5.5 Possible use of artificial miRNA for disease resistance

Resistance against a disease can also be achieved by artificial microRNA (miRNA)‐mediated knockdown of an S gene that facilitates infection. For instance, the dominant allele *Xa13*, which is responsible for susceptibility against bacterial blight in rice, was successfully knocked down using artificial miRNA in transgenic rice (Li, Wei, et al., 2012). In this study, tissue‐specific promoters were used, which included the promoter of the gene permitting expression of ribulose‐1,5‐bisphosphate carboxylase/oxygenase in leaves only, so that the artificial miRNA may not be expressed in other parts, including anthers (pollen development), so that *Xa13* functions normally in other parts, particularly during pollen development. Several highly resistant transgenic plants were obtained, which exhibited normal seed setting, suggesting that highly specific miRNA‐mediated gene silencing of S genes is possible to develop resistant cultivars.

## 6 CONCLUSIONS AND FUTURE PERSPECTIVES

Plant breeders develop disease‐resistant cultivars using a variety of approaches including pyramiding of R genes to provide durable resistance. The most important of these approaches is the use of dominant R genes, which follow gene‐for‐gene relationships. However, in recent years, several classes of dominant S genes have been discovered, so that the recessive alleles of these S genes are deployed for developing resistant cultivars. An important class of these S genes are those that are used by specific virulence genes in the pathogen causing the disease. One of the classes of these host S genes is the SWEET genes (encoding sugar transporters), whose dominant alleles are presumably used by the pathogens for supply of nutrition in the form of sugars. Another class of such sensitivity or S genes includes genes like *Tsn1*, which also follow inverse gene‐for‐gene relationships and are used by some necrotrophic pathogens causing wheat diseases like tan spot, spot blotch, and Stagonospora nodorum blotch. In this case also (as in case of SWEET genes), the plant–pathogen interaction depends upon the availability of a dominant S gene in the host.

SWEET genes have been identified in approximately 30 plant species, but they are only exploited in approximately 10 species as S genes by pathogens. Hopefully, more SWEET genes and species carrying these genes that are used by the pathogens will be discovered in the near future. Therefore, there is a need to continue the search for pathosystems involving SWEET genes functioning as S genes. In cases where SWEET genes are already known to function as S genes, there is a need to study the genetic variability of individual SWEET genes to (a) identify naturally occurring alternative recessive alleles for the dominant SWEET S genes, and (b) utilize them in crop breeding programmes for developing resistant cultivars. These SWEET genes carry binding sites in their promoters for a specific type of pathogen effectors called TALEs. TALENs and the 2020 Chemistry Nobel Prize winning approach CRISPR/Cas9 have also been used for genome editing, leading to the development of resistant genotypes.

The knowledge about the molecular basis of pathogenesis involving SWEET genes has also been used for designing strategies for developing disease‐resistant crops. These strategies will be the focus of attention in future plant breeding programmes and may include a number of approaches discussed in detail in this review. The most important example of the use of SWEET genes to achieve disease resistance is bacterial blight of rice. Two important papers and a commentary published recently (Eom et al., 2019; Oliva et al., 2019; Varshney et al., 2019), illustrate how the study and manipulation of SWEET genes can be utilized for developing bacterial blight‐resistant rice cultivars for different parts of the world. A diagnostic kit is also now available, which will facilitate the use of available information and knowledge to stimulate the development and deployment of resistant rice cultivars. However, bacterial blight in rice is not the only example. Several examples of diseases in a variety of crops are now available where SWEET genes are used by the pathogens to cause infection. Further work in these different crops at the same level at which bacterial blight in rice has been investigated will certainly help to find ways and means of developing disease resistance in a number of crops. The use of systems other than the classical R genes encoding NBS‐LRR proteins illustrates the significance of studies involving SWEET genes and their utilization. The ultimate objective of all these studies is the development of disease‐resistant cultivars not only in rice but also in other crops. A large number of bioinformatics tools and diagnostic kits of the kind developed in rice will also help in designing strategies for developing disease‐resistant cultivars.

Another important area of current and future research is the study of the origin and evolution of the T3SS, which is used by pathogens to secrete T3Es. The structure of the T3SS is known to play a crucial role in prokaryote–eukaryote interactions during infection, such that diversification of T3SSs in bacterial pathogens is also an important area of research; at least seven distinct T3SS families are already known (for a review, see Merda et al., 2017). The use of the T3SS also differs in pathogens for animal and plant cells. In a recent study, T3SSs and T3Es were examined in bacterial systems causing a variety of diseases in mammals (including humans), although no SWEET genes are involved in this system (Ruano‐Gallego et al., 2021). Therefore, it will also be interesting to compare the structures of T3SSs and the mechanisms involved in the secretion of T3Es using the T3SSs in pathogens causing diseases in plants and animals.

## Supporting information


**TABLE S1** Number of SWEET genes reported following genome‐wide studies in different plant species

**TABLE S2** Alternative genes used as targets by pathogens using TALEs

**TABLE S3** Bioinformatics tools for identification and classification of EBEs and TALEs and also for designing TALEs

**TABLE S4** A summary of studies conducted involving the use of natural/induced variations in SWEET genes to achieve disease resistance through loss of susceptibility in plants

**TABLE S5** A summary of natural variants in EBEs in the promoters of SWEET genes in rice

## Data Availability

Data sharing is not applicable to this article as no datasets were generated or analysed during the current study.
