# Supplementary material for: SWEET genes and TAL effectors for disease resistance in plants: Present status and future prospects
Source: Mol Plant Pathol. 2021 Jun 2;22(8):1014–26. doi: 10.1111/mpp.13075 (PMC8295518; doi:10.1111/mpp.13075)
Supplement: Supplementary file 1 — TABLE S1 Number of SWEET genes reported following genome‐wide studies in different plant species [file MPP-22-1014-s004.docx]

**SUPPLEMENTARY TABLE 1** Number of SWEET genes reported following genome-wide studies in different plant species.

| Species | Number of genes | Reference |
| --- | --- | --- |
| *Ananas comosus* | 39 | Guo et al., 2018 |
| *Arabidopsis thaliana* | 17 | Chen et al., 2010 |
| *Brassica napus* | 68 | Jian et al., 2016 |
| *Brassica oleracea* | 30 | Zhang et al., 2019 |
| *Brassica rapa* | 32, 33 | Li, Li, et al., 2018, Wei *et al*., 2019 |
| *Camellia sinensis* | 13 | Wang, Yao, et al., 2018 |
| *Citrus sinensis* | 16 | Zheng et al., 2014 |
| *Cucumis sativus* | 17, 17 | Hu et al., 2017, Li, Feng, et al., 2017 |
| *Eriobotrya japonica* | 7 | Wu et al., 2017 |
| *Glycine max* | 52 | Patil et al., 2015 |
| *Gossipiumarboreum/raimondii/hirsutum/barbadense* | 22, 31, 55, 60 | Zhao et al., 2018 |
| *Gossypium hirsutum* | 55 | Li, Ren, et al., 2018 |
| *Hevea brasiliensis* | 36 | Sui et al., 2017 |
| *Litchi chinensis* | 16 | Xie et al., 2019 |
| *Malus domestica* | 33 | Wei et al., 2014 |
| *Medicago truncatula* | 25 | Hu et al., 2019 |
| *Musa acuminate* | 25 | Miao et al., 2017 |
| *Oryza sativa* | 21 | Yuan & Wang, 2013 |
| *Phalaenopsis equestris*/*Dendrobium officinale* | 16, 22 | Wang, Song, et al., 2018 |
| *Pyrus bretschneideri* | 18 | Li, Qin, et al., 2017 |
| *Solanum lycopersicum* | 29, 23 | Feng et al., 2015 |
| *Solanum tuberosum* | 35 | Manck-Götzenberger & Requena, 2016 |
| *Sorghum bicolor* | 23 | Mizuno et al., 2016 |
| *Triticum aestivum* | 71, 108, 105 | Gao et al., 2018, Gautam et al., 2019, Qin et al., 2020 |
| *Vitis vinifera* | 17 | Chong et al., 2014 |
| *Medicago truncatula/ Pisum sativum* | 26, 22 | Doidy et al., 2019 |

**References**

Chen, L.-Q., Hou, B.-H., Lalonde, S., Takanaga, H., Hartung, M.L., Qu, X.-Q. et al. (2010) Sugar transporters for intercellular exchange and nutrition of pathogens. *Nature*, 468, 527–532.

Chong, J., Piron, M.C., Meyer, S., Merdinoglu, D., Bertsch, C. & Mestre, P. (2014) The SWEET family of sugar transporters in grapevine: *VvSWEET4* is involved in the interaction with *Botrytis cinerea*. *Journal of Experimental Botany*, 65, 6589–6601.

Doidy, J., Vidal, U. & Lemoine, R. (2019) Sugar transporters in Fabaceae, featuring SUT MST and SWEET families of the model plant *Medicago truncatula* and the agricultural crop *Pisum sativum*. *PloS One*, 14, e0223173.

Feng, C.Y., Han, J.X., Han, X.X. & Jiang, J. (2015) Genome-wide identification, phylogeny, and expression analysis of the SWEET gene family in tomato. *Gene*, 573, 261–272.

Gao, Y., Wang, Z.Y., Kumar, V., Xu, X.F., Yuan, P., Zhu, X.F. et al*.* (2018) Genome-wide identification of the SWEET gene family in wheat. *Gene*, 642, 284–292.

Gautam, T., Saripalli, G., Gahlaut, V., Kumar, A., Sharma, P.K., Balyan, H.S. et al*.* (2019) Further studies on sugar transporter (SWEET) genes in wheat (*Triticum aestivum* L.). *Molecular Biology Reports*, 46, 2327–2353.

Guo, C., Li, H., Xia, X., Liu, X. & Yang, L. (2018) Functional and evolution characterization of SWEET sugar transporters in *Ananas comosus*. *Biochemical and Biophysical Research Communications*, 496, 407-414.

Hu, B., Wu, H., Huang, W., Song, J., Zhou, Y. & Lin, Y. (2019) SWEET gene family in *Medicago truncatula*: genome-wide identification, expression and substrate specificity analysis. *Plants*, 8, 338.

Hu, L.P., Zhang, F., Song, S.H., Tang, X.W., Xu, H., Liu, G.-M. et al. (2017) Genome-wide identification, characterization, and expression analysis of the SWEET gene family in cucumber. *Journal of Integrative Agriculture*, 16, 1486-501.

Jian, H., Lu, K., Yang, B., Wang, T., Zhang, L., Zhang, A. et al*.* (2016) Genome-wide analysis and expression profiling of the SUC and SWEET gene families of sucrose transporters in oilseed rape (*Brassica napus* L.). *Frontiers in Plant Science*, 7, 1464.

Li, H., Li, X., Xuan, Y., Jiang, J., Wei, Y. & Piao, Z. (2018) Genome wide identification and expression profiling of *SWEET* genes family reveals its role during *Plasmodiophora brassicae*-Induced formation of clubroot in *Brassica rapa*. *Frontiers in Plant Science*, 9, 207.

Li, J., Qin, M., Qiao, X., Cheng, Y., Li, X., Zhang, H. et al*.* (2017) A new insight into the evolution and functional divergence of sweet transporters in Chinese white pear (*Pyrus bretschneideri*). *Plant Cell Physiology,* 58, 839–850.

Li, W., Ren, Z., Wang, Z., Sun, K., Pei, X., Liu, Y. et al*.* (2018) Evolution and stress responses of *Gossypium hirsutum* SWEET Genes. *International Journal of Molecular Sciences*, 19, 769.

Li, Y., Feng, S., Ma, S., Sui, X., & Zhang, Z. (2017) Spatiotemporal expression and substrate specificity analysis of the cucumber SWEET gene family. *Frontiers in Plant Science*, *8*, 1855.

Manck-Götzenberger, J. & Requena, N. (2016) Arbuscular mycorrhiza symbiosis induces a major transcriptional reprogramming of the potato SWEET sugar transporter family. *Frontiers in Plant Science*, 7, 487.

Miao, H., Sun, P., Liu, Q., Miao, Y., Liu, J., Zhang, K. et al*.* (2017) Genome-wide analyses of SWEET family proteins reveal involvement in fruit development and abiotic/biotic stress responses in banana. *Scientific Reports*, 7, 3536.

Mizuno, H., Kasuga, S. & Kawahigashi, H. (2016) The sorghum SWEET gene family: stem sucrose accumulation as revealed through transcriptome profiling. *Biotechnology for Biofuels*, 9, 127.

Patil, G., Valliyodan, B., Deshmukh, R., Prince, S., Nicander, B., Zhao, M. et al. (2015) Soybean (*Glycine max*) SWEET gene family: insights through comparative genomics, transcriptome profiling and whole genome re-sequence analysis. *BMC Genomics*, 16, 520.

[Qin,](https://www.sciencedirect.com/science/article/pii/S2095311919627619#!) J., [Jiang,](https://www.sciencedirect.com/science/article/pii/S2095311919627619#!) Y., [Lu,](https://www.sciencedirect.com/science/article/pii/S2095311919627619#!) Y., [Zhao,](https://www.sciencedirect.com/science/article/pii/S2095311919627619#!) P., [Wu,](https://www.sciencedirect.com/science/article/pii/S2095311919627619#!) B., [Li,](https://www.sciencedirect.com/science/article/pii/S2095311919627619#!) H. et al. (2020) Genome-wide identification and transcriptome profiling reveal great expansion of SWEET gene family and their wide-spread responses to abiotic stress in wheat (*Triticum aestivum* L.). [*Journal of Integrative Agriculture*](https://www.sciencedirect.com/science/journal/20953119),[19,](https://www.sciencedirect.com/science/journal/20953119/19/7) 1704-1720.

[Sui](https://febs.onlinelibrary.wiley.com/action/doSearch?ContribAuthorStored=Sui%2C+Jin-Lei), J.L., [Xiao](https://febs.onlinelibrary.wiley.com/action/doSearch?ContribAuthorStored=Xiao%2C+Xiao-Hu), X.H., [Qi](https://febs.onlinelibrary.wiley.com/action/doSearch?ContribAuthorStored=Qi%2C+Ji-Yan), J.Y., [Fang](https://febs.onlinelibrary.wiley.com/action/doSearch?ContribAuthorStored=Fang%2C+Yong-Jun), Y.J. & Tang, C.R. (2017) The SWEET gene family in *Hevea brasiliensis*– its evolution and expression compared with four other plant species. *FEBS Openbio*, 7, 1943-1959.

Wang, L., Yao, L., Hao, X., Li, N., Qian, W., Yue, C. et al*.* (2018) Tea plant SWEET transporters: expression profiling, sugar transport, and the involvement of *CsSWEET16* in modifying cold tolerance in Arabidopsis. *Plant Molecular Biology*, 96, 577–592.

Wang, T., Song, Z., Meng, W.L. & Li, L.B. (2018) Identification, characterization, and expression of the SWEET gene family in *Phalaenopsis equestris* and *Dendrobium officinale*. *Biologia Plantarum,* 62, 24–32.

Wei, X., Liu, F., Chen, C., Ma, F. & Li, M. (2014) The *Malus domestica* sugar transporter gene family: identifications based on genome and expression profiling related to the accumulation of fruit sugars. *Frontiers in Plant Science*, *5*, 569.

Wei, Y., Xiao, D., Zhang, C. & Hou, X. (2019) The expanded SWEET gene family following whole genome triplication in *Brassica rapa*. *Genes*, 10, 722.

Wu, Y., Wang, Y., Shan, Y. & Qin, Q. (2017) Characterization of SWEET family members from loquat and their responses to exogenous induction. *Tree Genetics & Genomes,* 13**,**123.

Xie, H., Wang, D., Qin, Y., Ma A., Fu, J., Qin, Y. et al*.* (2019) Genome-wide identification and expression analysis of *SWEET* gene family in *Litchi chinensis* reveal the involvement of *LcSWEET2a/3b* in early seed development. *BMC Plant Biology,* 19**,**499.

Yuan, M. & Wang, S. (2013) Rice MtN3/saliva/SWEET family genes and their homologs in cellular organisms. *Molecular Plant*, 6, 665-674.

Zhang, W., Wang, S., Yu, F., Tang, J., Shan, X., Bao, K. et al*.* (2019) Genome-wide characterization and expression profiling of SWEET genes in cabbage (*Brassica oleracea* var. *capitata* L.) reveal their roles in chilling and clubroot disease responses. *BMC Gnomics*, 20, 93.

Zhao, L., Yao, J., Chen, W., Li, Y., Lu, Y., Guo, Y. et al*.* (2018) A genome-wide analysis of *SWEET* gene family in cotton and their expressions under different stresses. *Journal of Cotton Research,* 1**,**7.

Zheng, Q., Tang, Z., Xu, Q. & Deng, X.X. (2014) Isolation, phylogenetic relationship and expression profiling of sugar transporter genes in sweet orange (*Citrus sinensis*). *Plant Cell Tissue and Organ Culture,* 119**,**609–624.
