# Supplementary material for: SWEET genes and TAL effectors for disease resistance in plants: Present status and future prospects
Source: Mol Plant Pathol. 2021 Jun 2;22(8):1014–26. doi: 10.1111/mpp.13075 (PMC8295518; doi:10.1111/mpp.13075)
Supplement: Supplementary file 2 — TABLE S2 Alternative genes used as targets by pathogens using TALEs [file MPP-22-1014-s001.docx]

**SUPPLEMENTARY TABLE 2**Alternative genes used as targets of the pathogen using TALEs

| Target | Disease | TALE | Reference |
| --- | --- | --- | --- |
| General transcription factors | | | |
| *OsTFIIAγ5* (small subunit of TFIIA) | Multiple diseases caused by Xanthomonas group | Multiple TALEs | Huang et al., 2017 |
| *OsTFIIAγ1*(small subunit of TFIIA) | Bacterial blight | PthXo7 | Sugio et al., 2007 |
| Specific transcription factors | | | |
| *OsTFX1* (bZIP TF) | Bacterial blight | PthXo6, TalB | Sugio et al., 2007; Tran et al., 2018 |
| *OsERF#123* (AP2/ERF TF) | Bacterial blight | TalB | Tran et al., 2018 |
| *CsLOB1* (TF) | Citrus bacterial canker | PthA4, PthAW, PthA* | Hu et al., 2014 |
| *UPA17* (GRF family TF) | Bacterial spot disease | AvrBs3 | Kay et al., 2009 |
| *UPA20* (bHLH family TF) | Bacterial spot disease | AvrBs3 | Kay et al., 2007, 2009 |
| Transporters | | | |
| *OsSULTR3;6* (Sulphate transporter) | Bacterial Leaf Streak | Tal2g | Cernadas et al., 2014 |
| *OsLsi1* (Silicon transporter) | Bacterial Leaf Streak | TalAL | Cernadas et al., 2014; Grau et al., 2013 |
| *OsPHO1;3* (Phosphate transporter) | Bacterial Leaf Streak | TalAO | Cernadas et al., 2014; Grau et al., 2013 |
| *OsNPF6.3* (Nitrate and auxin transporter) | Bacterial Leaf Streak | TalAE | Cernadas et al., 2014; Grau et al., 2013 |
| Phyto-hormones associated genes | | | |
| *UPA1-5* (Auxin-induced genes) | Bacterial spot disease | AvrBs3 | Marois et al., 2002 |
| *OsDOX-1* (2-oxoglutarate dioxygenase (SA)) | Bacterial Leaf Streak | TalAQ | Cernadas et al., 2014; Grau et al., 2013 |
| *TaNCED* (*9-cis-epoxycarotenoid dioxygenase* (ABA)) | Bacterial leaf streak | Tal8 | Peng et al., 2019 |
| Cell wall associated genes | | | |
| *OsPL* (Pectate lyase) | Bacterial leaf blight | TalAB | Mücke et al., 2019 |
| *OsWAK51* (Wall-associated kinase (WAK) receptor-like protein) | Bacterial leaf blight | TalES | Mücke et al., 2019 |
| *UPA6-7* (α-expansins) | Bacterial spot disease | AvrBs3 | Marois et al., 2002 |
| *UPA8* (Pectate lyase) | Bacterial spot disease | AvrBs3 | Marois et al., 2002 |
| *UPA15* (Mannan synthase) | Bacterial spot disease | AvrBs3 | Kay et al., 2009 |
| Secondary metabolites | | | |
| *UPA11* (Anthocyanidin rhamnosyl transferases) | Bacterial spot disease | AvrBs3 | Marois et al., 2002 |
| *UPA14* (Inositol 3-kinase) | Bacterial spot disease | AvrBs3 | Kay et al., 2009 |
| *OsFNS* (Flavone synthase) | Bacterial Leaf Streak | TalAQ | Cernadas et al., 2014 |
| Other genes with different functions | | | |
| *OsHEN1* (RNA methyltransferase) | Bacterial Leaf Streak | TalAP | Cernadas et al., 2014; Grau et al., 2013 |
| *OsHLS1* (Histone acetyltransferase | Bacterial leaf blight | TalBA | Mücke et al., 2019 |
| *OsFBX109* (Protein ubiquitination and protein degradation) | Bacterial leaf blight | TalAD | Mücke et al., 2019 |
| UPA21 (RNA‐binding protein) | Bacterial spot disease | AvrBs3 | Kay et al., 2009 |
| UPA22 (Light‐induced protein) | Bacterial spot disease | AvrBs3 | Kay et al., 2009 |

UPA: up-regulated by AvrBs3, ABA: abscisic acid, SA: salicylic acid

**References**

Cernadas, R.A., Doyle, E.L., Niño-Liu, D.O., Wilkins, K.E., Bancroft, T., Wang, L. et al*.* (2014) Code-assisted discovery of TAL effector targets in bacterial leaf streak of rice reveals contrast with bacterial blight and a novel susceptibility gene. *PLoS Pathogens,* 10, e1003972.

Grau, J., Wolf, A., Reschke, M., Bonas, U., Posch, S. & Boch, J. (2013) Computational predictions provide insights into the biology of TAL effector target sites. *PLoS Computational Biology*, 9, e1002962.

Hu, Y., Zhang, J., Jia, H., Sosso, D., Li, T., Frommer, W.B. et al. (2014) *Lateral organ boundaries 1* is a disease susceptibility gene for citrus bacterial canker disease. *Proceedings of the National Academy of Sciences of the United States of America*, 111, 521–529.

Huang, R., Hui, S., Zhang, M., Li, P., Xiao, J., Li, X. et al. (2017) A conserved basal transcription factor is required for the function of diverse TAL effectors in multiple plant hosts. *Frontiers in Plant Science*, 8:1919.

Kay, S., Hahn, S., Marois, E., Hause, G. & Bonas, U. (2007) A bacterial effector acts as a plant transcription factor and induces a cell size regulator. *Science*, 318, 648–651.

Kay, S., Hahn, S., Marois, E., Wieduwild, R. & Bonas, U. (2009) Detailed analysis of the DNA recognition motifs of the *Xanthomonas* type III effectors AvrBs3 and AvrBs3Deltarep16. *The Plant Journal*, 59, 859–871.

Marois, E., Van den Ackerveken, G. & Bonas, U. (2002) The *Xanthomonas* type III effector protein AvrBs3 modulates plant gene expression and induces cell hypertrophy in the susceptible host. *Molecular Plant-Microbe Interactions*, 15, 637–646.

Mücke, S., Reschke, M., Erkes, A., Schwietzer, C.A., Becker, S., Streubel, J. et al. (2019) Transcriptional reprogramming of rice cells by *Xanthomonas oryzae* TALEs. *Frontiers in Plant Science*, 10, 162.

Peng, Z., Hu, Y., Zhang, J., Huguet-Tapia, J.C., Block, A. K., Park, S. et al. (2019) *Xanthomonas translucens* commandeers the host rate-limiting step in ABA biosynthesis for disease susceptibility. *Proceedings of the National Academy of Sciences of the United States of America*, 116, 20938-20946.

Sugio, A., Yang, B., Zhu, T. & White, F.F. (2007) Two type III effector genes of *Xanthomonas oryzae* pv. *oryzae* control the induction of the host genes *OsTFIIAgamma1* and *OsTFX1* during bacterial blight of rice. *Proceedings of the National Academy of Sciences of the United States of America*, 104, 10720-10725.

Tran, T.T., Pearez-Quintero, A.L., Wonni, I., Carpenter, S.C.D., Yu, Y., Wang, L. et al. (2018) Functional analysis of African *Xanthomonas oryzae* pv. *oryzae* TALomes reveals a new susceptibility gene in bacterial leaf blight of rice. *PLoS Pathogen*, 14, e1007092.
