# Supplementary material for: SWEET genes and TAL effectors for disease resistance in plants: Present status and future prospects
Source: Mol Plant Pathol. 2021 Jun 2;22(8):1014–26. doi: 10.1111/mpp.13075 (PMC8295518; doi:10.1111/mpp.13075)
Supplement: Supplementary file 3 — TABLE S3 Bioinformatics tools for identification and classification of EBEs and TALEs and also for designing TALEs [file MPP-22-1014-s003.docx]

**SUPPLEMENTARY TABLE 3** Bioinformatics tools for identification and classification of EBE and TALEs and also for designing TALEs.

| Tool | Method | Purpose | Reference |
| --- | --- | --- | --- |
| Target Finder from TALE-NT2.0 suit  * | Prediction of EBE relies on the use of RVD-nucleotide association matrix to convert a sequence of RVDs of a given TALE into positional weight matrix (PWM) | Scan genomes for TALE binding elements (EBE), provides a list of potential S and R targets and identifying potential off-target sites | Doyle et al., 2012 |
| Talvez*, Storyteller* | Predictions by Talvez also rely on the conversion of sequence of RVDs of a given TALE into PWM.  Storyteller, on the other hand, generates a set of possible binding sequences and takes advantage of Hidden Markov Models. | Scan genomes for TALEbinding elements (EBE) and provides a list of potential S and R targets | Pérez-Quintero et al., 2013 |
| TALgetter* | Based on a statistical model for which parameters are estimated from training data computationally; uses local mixture model | Scan genomes for TALE binding elements (EBE) and provides a list of potential S and R targets | Grau et al., 2013 |
| SIFTED  (webserver is currently unavailable) * | Custom-designed custom-protein-binding microarrays (PBMs) to assay TALE–DNA-binding specificity | Prediction of DNA binding specificity and off-target sites for improved TALE designing | Rogers et al., 2015 |
| DisTAL  and FuncTALprogrammes in QueTAL suit* | Custom designed; use Perl modules Statistics::R, Bio::Perl, and the R library APE | Identification of TALE genes; classification of TALEs according to phylogeny and with similar DNA-binding specificities | Pérez-Quintero et al., 2013, 2015 |
| AnnoTALE  (a suite of applications) ** | Local mixture model | Prediction, analysis, grouping, and nomenclature of TALE genes from *X*. *oryzae*genome sequences and prediction of EBEs | Grau et al., 2016 |
| PrediTALE* | Use quantitative data and previous information. | Prediction of TALE targets and identification of novel putative virulence targets | Erkes et al., 2019 |
| TargeTALE  webserver** | Developed using Python language and third party packages BioPython, Bioservices, and Requests | Can identify TALE genes and also their targets along with functional annotation of the identified targets using Gene Ontology (GO) terms. | Kremer et al., 2019 |

*predicts only EBEs; **predicts both EBEs and TALEs

**References**

Doyle, E.L., Booher, N.J., Standage, D.S., Voytas, D.F., Brendel, V.P., Vandyk, J.K. et al*.* (2012) TAL Effector-Nucleotide Targeter (TALE-NT) 2.0: tools for TAL effector design and target prediction. *Nucleic Acids Research*, 40, 117-122.

Pérez-Quintero, A.L., Rodriguez-R, L.M., Dereeper, A., López, C., Koebnik, R., Szurek, B. et al. (2013) An improved method for TAL effectors DNA-binding sites prediction reveals functional convergence in TAL repertoires of *Xanthomonas oryzae* strains. *PloS One*, 8, e68464.

Grau, J., Wolf, A., Reschke, M., Bonas, U., Posch, S. & Boch, J. (2013) Computational predictions provide insights into the biology of TAL effector target sites. *PLoS Computational Biology*, 9, e1002962.

Rogers, J.M., Barrera, L.A., Reyon, D., Sander, J.D., Kellis, M., Joung, J.K. et al*.* (2015) Context influences on TALE-DNA binding revealed by quantitative profiling. *Nature Communications*, 6, 7440.

Pérez-Quintero, A.L., Lamy, L., Gordon, J.L., Escalon, A., Cunnac, S., Szurek, B. et al*.* (2015) QueTAL: a suite of tools to classify and compare TAL effectors functionally and phylogenetically. *Frontiers in Plant Science*, 6, 545.

Grau, J., Reschke, M., Erkes, A., Streubel, J., Morgan, R.D., Wilson, G.G. et al*.* (2016) AnnoTALE: bioinformatics tools for identification, annotation and nomenclature of TALEs from *Xanthomonas* genomic sequences. *Science Reports,* 6, 21077.

Erkes, A., Mücke, S., Reschke, M., Boch, J. & Grau, J. (2019) PrediTALE: A novel model learned from quantitative data allows for new perspectives on TALE targeting. *PLoS Computational Biology*, 15, e1007206.

Kremer, F.S., Guimarães, A.M., Sanchez, C.D. & da Silva Pinto, L. (2019) TargeTALE: A web resource to identify TALEs in *Xanthomonas* genomes and their respective targets. *Molecular Plant-Microbe Interaction*, 32, 1577-1580.
