# Supplementary material for: SWEET genes and TAL effectors for disease resistance in plants: Present status and future prospects
Source: Mol Plant Pathol. 2021 Jun 2;22(8):1014–26. doi: 10.1111/mpp.13075 (PMC8295518; doi:10.1111/mpp.13075)
Supplement: Supplementary file 4 — TABLE S4 A summary of studies conducted involving the use of natural/induced variations in SWEET genes to achieve disease resistance through loss of susceptibility in plants [file MPP-22-1014-s005.docx]

**SUPPLEMENTARY TABLE 4** A summary of studies conducted involving the use of natural/induced variations in SWEET genes for achieving disease resistance through loss of susceptibility (RLS) in plants.772383003571

| Crop, disease & pathotypes/TALE | SWEET gene | Method | Reference |
| --- | --- | --- | --- |
| Rice (BB^$^), PthXo1 | *OsSWEET11= xa13* (*Os8N3*) | Natural sequence polymorphism in EBE | See review by Schornack et al., 2013 |
| Rice (BB); PthXo2,3; AvrXa7 | *OsSWEET13, 14* (*xa25, xa41*) | Natural variants in EBE (deletion of 5, 6 and substitution of 10, 17bases in EBE) | Zaka et al., 2018 |
| Rice (BB), AvrXa27 | *-* | Trap promoter in *Xa27* | Gu et al., 2005 |
| Pepper *Xeu* Euves.* AvrBs3*, X. gardneri*AvrHah1 (BS^#^) |  | Trap promoter in *Bs3* gene | Schornack et al., 2005, |
| Rice (BB), AvrXa7, PthXo3 | *Os11N3 = OsSWEET14 = xa41* | TALEN gene editing | Li, Liu, et al., 2012 |
| Rice AvrXa7, Tal5 or TalC | *OsSWEET11* | TALEN gene editing | Blanvillain-Baufume et al., 2017 |
| Rice (BB) | *OsSWEET14* | CRISPR/Ca9 | Jiang et al., 2013 |
| Rice (BB) OsSWEET11 |  | CRISPR/Cas9 | Zhou et al., 2014 |
| Rice Kitaake (BB) PthXo1,PthXo3/AvrXa7 | *OsSWEET11, 14* | CRISPR/Cas9 editing of EBE | Xu et al., 2019 |
| Rice (BB) Kitaake, IR64, Ciherang-Sub1 | *OsSWEET11*, *13, 14* | CRISPR/Cas9 | Oliva et al., 2019 |
| Rice (BB), Kitaake, |  | CRISPR/Cas9 | Eom et al., 2019 |
|  | *OsSWEET11* | RNAi | Yang et al., 2006 |

^$^BB= Bacterial blight; **Xeu = X. euvesicatoria*; ^#^*BS*= Bacterial spot

**References**

Blanvillain-Baufumé, S., Reschke, M., Solé, M., Auguy, F., Doucoure, H., Szurek, B. et al. (2017) Targeted promoter editing for rice resistance to *Xanthomonas oryzae* pv. *oryzae* reveals differential activities for *SWEET14*-inducing TAL effectors. *Plant Biotechnology Journal*, 15, 306–317.

Eom, J., Luo, D., Atienza-Grande, G., Yang, J., Ji, C., Luu, V.T. et al*.* (2019) Diagnostic kit for rice blight resistance. *Nature Biotechnology,* 37, 1372-1379.

Gu, K., Yang, B., Tian, D., Wu, L., Wang, D., Sreekala, C. et al*.* (2005) R gene expression induced by a type-III effector triggers disease resistance in rice. *Nature*, 435, 1122-1125.

Jiang, W., Zhou, H., Bi, H., Fromm, M., Yang, B. & Weeks, D.P. (2013) Demonstration of CRISPR/Cas9/sgRNA-mediated targeted gene modification in Arabidopsis, tobacco, sorghum and rice. *Nucleic Acids Research*, 41, e188.

Li, T., Liu, B., Spalding, M. H., Weeks, D. P. & Yang, B. (2012) High-efficiency TALEN-based gene editing produces disease-resistant rice. *Nature Biotechnology*, *30*, 390–392.

Oliva, R., Ji, C., Atienza-Grande, G., Huguet-Tapia, J.C., Perez-Quintero, A., Li, T. et al. (2019) Broad-spectrum resistance to bacterial blight in rice using genome editing. *Nature Biotechnology*, 37, 1344–1350.

Schornack, S., Moscou, M.J., Ward, E.R. & Horvath, D.M. (2013) Engineering plant disease resistance based on TAL effectors. *Annual Review of Phytopathology*, 51, 383-406.

Schornack, S., Peter, K., Bonas, U. & Lahaye, T. (2005) Expression levels of avrBs3-like genes affect recognition specificity in tomato Bs4- but not in pepper Bs3-mediated perception. *Molecular Plant-Microbe Interaction*, 18, 1215–1225.

Xu, Z., Xu, X., Gong, Q., Li, Z., Li, Y., Wang, S. et al*.* (2019) Engineering broad-spectrum bacterial blight resistance by simultaneously disrupting variable TALE-binding elements of multiple susceptibility genes in rice. *Molecular Plant*, 12, 1434–1446.

Yang, B., Sugio, A. & White, F.F. (2006) *Os8N3* is a host disease-susceptibility gene for bacterial blight of rice. *Proceedings of National Academy of Science of the United States of America*, 103, 10503–10508.

Zaka, A., Grande, G., Coronejo, T., Quibod, I.L., Chen, C.W., Chang, S.J., Szurek, B. et al. (2018) Natural variations in the promoter of *OsSWEET13* and *OsSWEET14* expand the range of resistance against *Xanthomonas oryzae* pv. *oryzae*. *PloS One*, 13, e0203711.

Zhou, Y., Liu, L., Huang, W., Yuan, M., Zhou, F., Li, X. et al. (2014) Overexpression of *OsSWEET5* in rice causes growth retardation and precocious senescence. *PLoS One*, 9, e94210.
