# Supplementary material for: SWEET genes and TAL effectors for disease resistance in plants: Present status and future prospects
Source: Mol Plant Pathol. 2021 Jun 2;22(8):1014–26. doi: 10.1111/mpp.13075 (PMC8295518; doi:10.1111/mpp.13075)
Supplement: Supplementary file 5 — TABLE S5 A summary of natural variants in EBEs in the promoters of SWEET genes in rice [file MPP-22-1014-s002.docx]

**TABLE S5** A summary of natural variants in EBE in the promoters of SWEET genes in rice.

| Repeat | 9 | 10 | 11 | Repeat | 4 | 5 | 6 | 7 | 8……..17 |
| --- | --- | --- | --- | --- | --- | --- | --- | --- | --- |
| PthXo3 RVDs | NN | HD | HD | PthXo2.1 RVDs | NN | NN | NI | NN | HD….HG |
| PthXa7 RVDs | HD | HD | NS |  |  |  |  |  |  |
| TBE |  |  |  |  |  |  |  |  |  |
| IR24 | C | C | T | IR24 | A | A | A | G | G……..T |
| Nipponbare | C | C | T | Mingui63 | A | A | - | G | G……..T |
| Ejali | C | **G** | T | ZS97 | A | - | - | G | G……..T |
| Kharra1183 | C | **G** | T | SB | A | A | - | G | G……..**A** |

Bold nucleotides at positions 10 and 17 indicate variable sites in EBE
